# Studying the Influence of Finite Element Mesh Size on the Accuracy of Ventricular Tachycardia Simulation

**DOI:** 10.31083/j.rcm2412351

**Published:** 2023-12-13

**Authors:** Boyang Cao, Nan Zhang, Zhenyin Fu, Ruiqing Dong, Tan Chen, Weiguo Zhang, Lv Tong, Zefeng Wang, Mingxia Ma, Zhanchun Song, Fuzhi Pan, Jinghui Bai, Yongquan Wu, Dongdong Deng, Ling Xia

**Affiliations:** ^1^College of Biomedical Engineering & Instrument Science, Zhejiang University, 310058 Hangzhou, Zhejiang, China; ^2^School of Biomedical Engineering, Dalian University of Technology, 116024 Dalian, Liaoning, China; ^3^Department of Radiology, Beijing Anzhen Hospital Affiliated to Capital Medical University, 100029 Beijing, China; ^4^Department of Radiology, Dushu Lake Hospital Affiliated to Soochow University, 215000 Suzhou, Jiangsu, China; ^5^Department of Cardiology, Beijing Anzhen Hospital Affiliated to Capital Medical University, 100029 Beijing, China; ^6^Department of General Medicine, Liaoning Cancer Hospital of Dalian University of Technology, 116024 Dalian, Liaoning, China; ^7^Department of Cardiology, Fushun Central Hospital, 113006 Fushun, Liaoning, China; ^8^Research Center for Healthcare Data Science, Zhejiang Lab, 310003 Hangzhou, Zhejiang, China

**Keywords:** ventricular tachycardia, computational modeling, mesh resolution, conduction velocity, tetrahedral meshes

## Abstract

**Background:**

Ventricular tachycardia (VT) is a life-threatening heart condition commonly seen in patients with myocardial infarction (MI). Although personalized computational modeling has been used to understand VT and its treatment noninvasively, this approach can be computationally intensive and time consuming. Therefore, finding a balance between mesh size and computational efficiency is important. This study aimed to find an optimal mesh resolution that minimizes the need for computational resources while maintaining numerical accuracy and to investigate the effect of mesh resolution variation on the simulation results.

**Methods:**

We constructed ventricular models from contrast-enhanced magnetic resonance imaging data from six patients with MI. We created seven different models for each patient, with average edge lengths ranging from 315 to 645 µm using commercial software, Mimics. Programmed electrical stimulation was used to assess VT inducibility from 19 sites in each heart model.

**Results:**

The simulation results in the slab model with adaptive tetrahedral mesh (same as in the patient-specific model) showed that the absolute and relative differences in conduction velocity (CV) were 6.1 cm/s and 7.8% between average mesh sizes of 142 and 600 µm, respectively. However, the simulation results in the six patient-specific models showed that average mesh sizes with 350 µm yielded over 85% accuracy for clinically relevant VT. Although average mesh sizes of 417 and 478 µm could also achieve approximately 80% accuracy for clinically relevant VT, the percentage of incorrectly predicted VTs increases. When conductivity was modified to match the CV in the model with the finest mesh size, the overall ratio of positively predicted VT increased.

**Conclusions:**

The proposed personalized heart model could achieve an optimal balance between simulation time and VT prediction accuracy when discretized with adaptive tetrahedral meshes with an average edge length about 350 µm.

## 1. Introduction

Ventricular tachycardia (VT) is a life-threatening heart condition frequently observed in patients with myocardial infarction (MI) and is a primary cause of sudden cardiac death (SCD) [1, 2]. Currently, catheter ablation is the standard treatment for VT, as medication has limited effectiveness [3, 4]. However, the success rate of ablation is low [5, 6]. As identifying the critical conduction pathways for reentry is challenging due to the limited spatial sampling of electrophysiological markers, poor correlation with cardiac anatomy, and potential complexity of the pathway as electrical excitation propagates, meaning favorable outcomes are yet to be achieved [7, 8, 9, 10, 11].

Personalized computational models based on finite element methods (FEM) and three dimensional (3D) cardiac geometry reconstructed from clinical imaging data (e.g., magnetic resonance imaging (MRI), computed tomography) have been proposed to simulate cardiac electrophysiology to identify critical conduction pathways [12, 13, 14, 15]. These models are employed in noninvasive studies of lethal arrhythmia and its treatments, such as risk stratification of MI patients, prediction of reentry location [5, 6, 16], and optimization of VT ablation [17]. However, individual computational modeling of VT is time consuming, often requiring less than 48 hours of simulation time, making its clinical application in guiding VT ablation challenging [12]. Additionally, obtaining reliable simulation results requires high-performance clusters (over 1000 cores for each patient simulation [12, 14]) and proper mesh resolution.

Previous theoretical studies have indicated that a mesh resolution of 0.25 mm or 0.1 mm is required in cardiac electrophysiological simulations to achieve below 10% simulation error [18, 19]. Numerous efforts have been made to enhance the accuracy and efficiency of cardiac electrophysiology simulation, including high-order finite element solvers [20, 21, 22], stabilization schemes for conduction velocity (CV) [23], or a modified quadrature approach [24]. Although these methods have been shown to improve the accuracy of cardiac electrophysiology simulation, first-order finite element solvers and tetrahedral mesh are still widely used for personalized heart modeling [12, 13, 25] using average mesh resolution ranging from 350 to 500 µm or even 1 mm [12, 25, 26, 27, 28, 29].

The mesh resolution in personalized heart models is significantly higher than those used for accuracy and convergence studies in cardiac electrophysiological simulations. Limited standardized spatial discretization methods exist to determine the optimal mesh resolution that best balances computational stability, efficiency, and reliability of results in personalized VT simulations. To address this gap, our study aims to comprehensively analyze the effects of mesh resolution on simulation results and identify an optimal mesh resolution for personalized computational modeling of VT.

## 2. Materials and Methods

### 2.1 Clinical Data

In this study, we recruited six MI patients between 2018 and 2020 from Beijing Anshan Hospital and Dushuhu Hospital of Suzhou University. The study was approved by the Institutional Review Board of both hospitals. Cardiac magnetic resonance–late gadolinium enhancement (CMR–LGE) images of the patients were used to construct the heart models. A cardiac MRI was acquired using a 3.0 T scanner. Detailed image acquisition protocol has been published previously [30, 31, 32]. Table 1 provides detailed information on the images for each patient.

**Table 1.** **Details of the CMR–LGE images of each patient**.

Patient	Size	In-plane resolution (mm)	Slice thickness (mm)	Scanning slices
PAT01	256 × 208	1.52344 × 1.52344	9	14
PAT02	256 × 256	1.6406 × 1.6406	8	10
PAT03	208 × 256	1.5625 × 1.5625	8	13
PAT04	240 × 256	1.52344 × 1.52344	10	10
PAT05	208 × 256	1.48438 × 1.48438	8	11
PAT06	256 × 192	1.36719 × 1.36719	8.4	10

CMR–LGE, cardiac magnetic resonance–late gadolinium enhancement.

### 2.2 Image Processing Pipeline

All analyses and measurements were performed using a custom software developed in MATLAB (Version: 2021a, Mathworks Inc., Natick, MA, USA). Two experienced experts manually segmented the epicardial and endocardial boundaries of the LGE images. The pixels between the boundaries were considered the myocardium. Then the modified Gaussian mixture model method was utilized to automatically identify the infarcted regions [33]. Finally, the full width at half max method was employed to further segment the infarcted tissue into the gray zone and core scar. The detailed process has been published previously [13, 33]. Following image segmentation, CardioViz3D [34] (INRIA, Sophia Antipolis, France) was used to interpolate the segmented low-resolution images to high-resolution images (approximately 0.4 mm). Then, the infarct tissue, including the core scar and gray zone, was interpolated using the log-odds method [35].

### 2.3 Model Generation with Different Resolutions

To quantitatively assess the impact of mesh resolution on the accuracy of electrical propagation in monodomain simulations, we introduced a cuboid of the same size used in the N-version benchmark [19] test. Table 2 provides detailed information about this cuboid, whose dimensions were 20 × 7 × 3 mm. Two different discretization methods were employed, which included uniform and adaptive tetrahedral meshes generated using Mesher in OpenCARP (opencarp.org) and 3-matic software (Materialize NV, Leuven, Belgium), respectively. As the adjustable parameters for different mesh resolutions varied between Mesher (shortest edge length) and 3-matic (maximal edge length), the mesh sizes for both discretization methods were different. The average mesh size of both meshes ranged from approximately 100 µm to 700 µm. Tables 3,4 (Ref. [19]) show the mesh statistics of both discretization methods.

**Table 2.** **Model-specific parameters**.

Variable	Description		
Geometric domain	Cuboid	Cable	Slab
Dimensions	20 mm × 7 mm × 3 mm	10 mm × 1 elem × 1 elem	20 mm × 20 mm × 3 mm
Mesh type	uniform/adaptive	uniform	adaptive
Fiber orientation	along the long axis	along the long axis	along the long axis
PDE time steps	10 µs/25 µs	25 μs	25 μs
Stimulation geometry	1.5 × 1.5 × 1.5 mm cube from a corner	1.0 mm × 1 elem × 1 elem cube from the start of the long axis	1.0 × 1.0 × 3 mm cube from the center
Intra-longitudinal, Intra-transversal	0.17, 0.019	0.08, 0.00889	0.08, 0.00889
Extra-longitudinal, Extra-transversal	0.625, 0.236	0.625, 0.236	0.625, 0.236
Cell model	ten Tusscher 2006	ten Tusscher 2006	ten Tusscher 2006
Mass lumping	Yes/No	No	No

PDE, partial differential equations; elem, element.

**Table 3.** **Mesh statistics and the activation times at the P8 point with uniform tetrahedral mesh, which showed in Fig. 1 (Ref. [19])**.

Uniform tetrahedral mesh (µm)	Mesh Statistics (mean ± std, µm)	Activation Time (ms)
90	108.5 ± 18.6	45.1
100	120.5 ± 20.7	44.5
200	240.7 ± 41.4	44.7
300	360.6 ± 62.1	48.1
350	420.4 ± 72.4	52.1
400	480.2 ± 82.8	58.0
500	599.3 ± 103.5	60.5
600	718.2 ± 124.1	73.1

The assumed converged solution at P8 was 43 ms based on reference [19].

**Table 4.** **Mesh statistics and the activation times at the P8 point with adaptive tetrahedral mesh, which showed in Fig. 1**.

Adaptive tetrahedral mesh (µm)	Mesh Statistics (mean ± std, µm)	Activation Time (ms)
140	100.3 ± 20.4	44.6
200	144.3 ± 30.2	43.4
300	219.2 ± 49.6	42.2
400	296.8 ± 71.5	41.7
500	375.0 ± 91.4	41.8
600	456.4 ± 118.5	41.7
700	538.1 ± 134.9	42.7
800	626.3 ± 159.2	43.6
900	716.2 ± 189.1	45.1

The assumed converged solution at P8 was 43 ms based on reference [19].

First, we used the N-version benchmark to verify the accuracy of parameters used for both discretization methods. Then, we used a 10 mm cable and a slab with dimensions of 20 × 20 × 3 mm to examine the relationship between CV and mesh resolution. Table 2 provides the details of these three models.

To analyze the effect of varying model resolutions on the simulation results of patient-specific models, we generated six different models for each patient using Mimics (Materialize NV, Leuven, Belgium). Each model’s maximum and average edge length ranged from 0.45–1 mm and 0.3–0.65 mm, respectively. The upper limit of the model resolution was selected based on the previous benchmark studies of numerical convergence in cardiac electrophysiological modeling [19] and to test the effect of model resolution outside the theoretical convergence range on patient-specific ventricular model simulation results. Whereas its lower limit was selected based on the computer resources for model generation and high-preformation cluster.

### 2.4 Electrophysiological Properties and Stimulation Protocols

After establishing the models, the fiber direction was specified in the mesh using a rule-based method [36]; The electrophysiological properties were assigned according to our previously published paper [13, 14]. Briefly, non-infarcted tissue was assigned to the human ventricular cell model published by ten Tusscher *et al*. [37]. Action potential remodeling in the gray zone, including changes in properties of fast delayed rectifier potassium, delayed rectifier potassium, L-type calcium, and sodium channels, was represented by reducing the corresponding currents ($I_{\text{Kr}}$, $I_{\text{Ks}}$, $I_{\text{CaL}}$, and $I_{\text{Na}}$) to 30%, 20%, 31%, and 38% of the normal values of the ten Tusscher ventricular myocyte model, based on previous experimental records [8, 38]. The core scar was modeled as an insulator that does not conduct electrical excitation. As shown in Table 2, the conductivities used for the cuboid were the same as in [19], but those for the cable, slab, and patient-specific model were set to match the clinically measured CV [10, 16, 39, 40, 41, 42].

The propagation of electrical activity in the heart model was simulated by solving a reaction-diffusion partial differential equation using FEM [43]. Electrical stimulations were performed using the openCARP simulation environment [44] on high-performance computers at the Dalian University of Technology, China, which was used to induce VTs as published previously [13]. All models were paced from 19 ventricular sites, including 17 sites on the left ventricle (LV), 1 near the right ventricular outflow tract, and 1 at the right ventricle apex, following the American Heart Association (AHA) Classification Standards [45]. After inducing reentry, arrhythmia was detected using a 10-second VT simulation based on the scheme proposed by Tong *et al*. [13].

### 2.5 Measuring and Adjusting the CV

The CV in the cable and slab simulations was calculated by taking the difference in activation times at locations 2.5 mm and 7.5 mm and 3.5 mm and 7.5 mm, respectively, and dividing it by the distance between the two points. CV values were calibrated by simulating wavefront propagation following stimulation at the center of a slab model (20 mm × 20 mm × 3 mm) with longitudinal fiber orientations along the X-axis. To achieve the same CV in models with different mesh resolutions, conductivities in all models were adjusted to match the CV of the highest-resolution model.

The VT locations in the clinic were measured as part of the electrophysiological study performed during implantable cardioverter defibrillator (ICD) implantation or estimated by a senior electrophysiologist via electrocardiogram (ECG). To compare the locations quantitatively via clinical assessment and simulation, the VT locations were assigned to the 17 segments following the American Heart Association Classification [45]. If the segment of the predicted VT location in the simulation was the same as the clinical measurement, it was considered clinically relevant reentry, or otherwise it was treated as a clinically irrelevant VT.

## 3. Results

### 3.1 Validation of Numerical Accuracy of the Different Mesh Types and Resolution

**Fig. 1.**
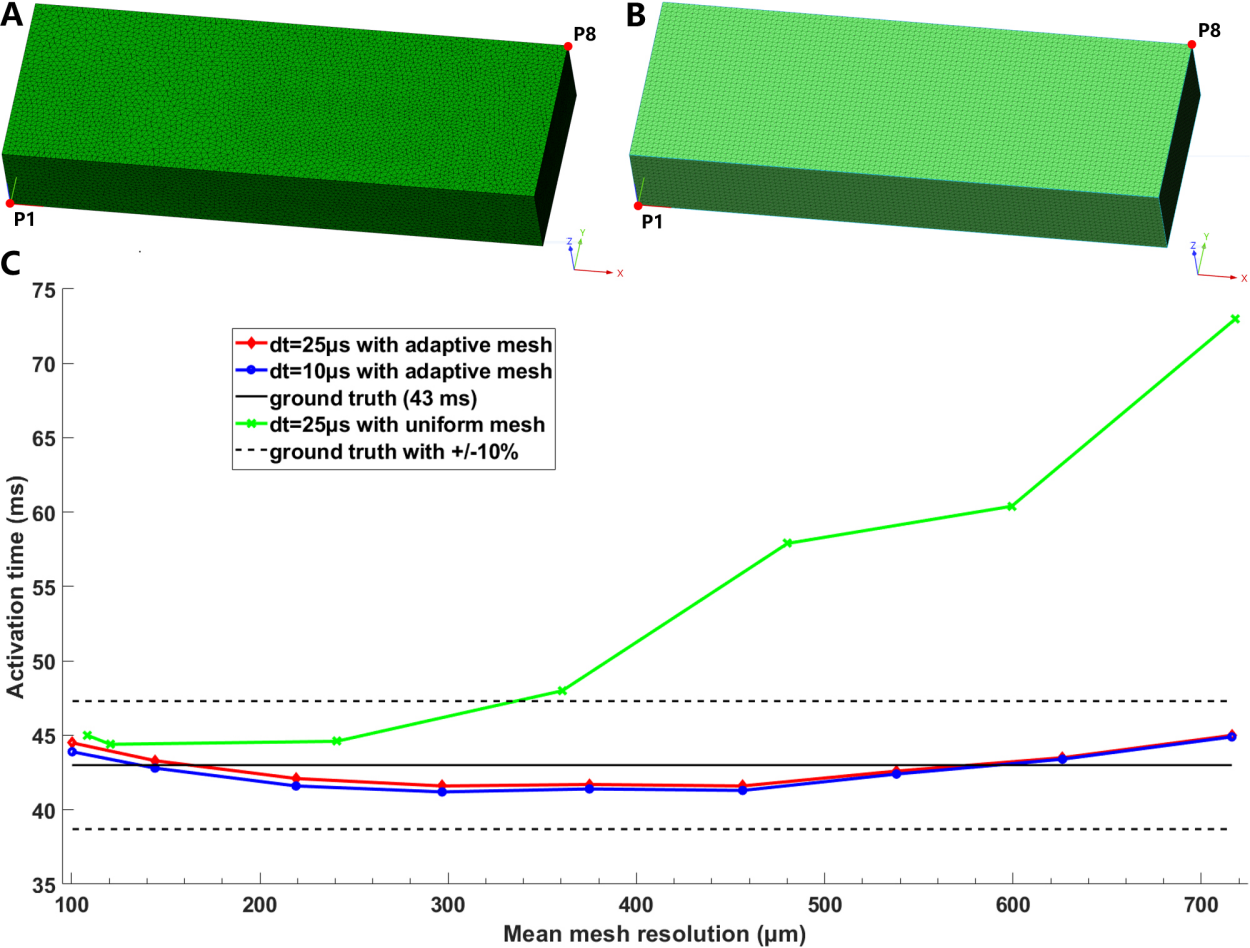
**Model discretization with adaptive (A) and uniform tetrahedral (B) mesh**. Activation times at point P8 for solutions with dt = 25 µs and 10 µs for adaptive meshes, and dt = 25 µs for uniform meshes (C). The ground truth of 43 ms is the assumed converged solution, as reported in reference [19].

Fig. 1C displays the activation times at point P8 for solutions with different time steps for adaptive and uniform meshes of the cuboid (Fig. 1A,B). The activation time at point P8 with a uniform mesh had below 10% error at a spatial scale of approximately 340 µm (green line in Fig. 1). When a spatial scale of uniform mesh size of 600 µm (718.2 ± 124.1 µm) was used (Table 3), the error was 65.1% compared to the assumed converged solution of 43 ms as reported in reference [19]. The activation time displayed nonmonotonic behavior when the mesh resolution was increased to 90 µm (108.5 ± 18.6 µm).

For simulations with adaptive meshes (red and blue lines in Fig. 1C), we observed minor differences between time steps of 25 and 10 µs. The error in the relative difference in activation time at P8 with adaptive tetrahedral meshes was within 10% compared to the assumed converged solution of 43 ms [19], although the simulation results with adaptive meshes also displayed nonmonotonic behavior when the mesh resolution was changed from an average value of 700 µm to 100 µm.

### 3.2 Meshes Statistics in Six Subjects

Table 5 summarizes the volumes of normal myocardium, gray zone, and core scar of the reconstructed cardiac models. The average ventricular volume was 174.3 ${\mathrm{cm}}^{3}$. The percentage of myocardial volume in non-infarcted areas and the gray zone was approximately 88.5%–96.9% and 2.5%–8.7%, respectively, with the core scar comprising 0.5%–4.9%. The variation in volume for each tissue (non-infarct tissue, gray zone (GZ), and core scar) in the models with different resolutions was within 0.01 ${\mathrm{cm}}^{3}$.

**Table 5.** **Summary of the volume database for six patients **.

ID	Non-infarct tissue (${\mathrm{cm}}^{3}$)	% of the total volume	Gray zone (${\mathrm{cm}}^{3}$)	% of the total volume	Core scar (${\mathrm{cm}}^{3}$)	% of the total volume
PAT01	208.1	94.7	6.7	3.1	4.9	2.2
PAT02	121.5	96.2	1.3	1.0	3.5	2.8
PAT03	178.0	87.8	10.4	5.1	14.3	7.1
PAT04	231.4	96.8	5.8	2.4	1.9	0.8
PAT05	104.2	87.3	9.0	7.6	6.1	5.1
PAT06	164.1	93.8	6.6	3.8	4.3	2.4
Mean ± std	161.6 ± 48.9	92.7 ± 4.2	5.6 ± 3.1	3.2 ± 2.3	4.8 ± 4.4	2.7 ± 2.2

Table 6 presents the statistical characteristics of the generated tetrahedral models with different mesh resolutions for all six patients. Res1–Res6 in the first column represent different model resolutions; the second column displays the value of the maximal edge length of the tetrahedral mesh for each model in the Mimics software; the third and fourth columns show the average number of vertices and elements for all six tetrahedral models with the corresponding resolution, respectively; the fifth column represents the average edge lengths for all six models with different maximum edge lengths; and the last column shows the simulation time for each time step of 10 ms.

**Table 6.** **Summary of the average mesh information for all six patients with different mesh resolutions**.

Model resolution	Maximal edge length in Mimics (µm)	Vertices (Million)	Elements (Million)	Edge Length (Mean ± std, µm)	Simulation time per 10 ms (unit: second)
Res1	1000	1.3 ± 0.5	6.7 ± 2.2	648.7 ± 19.8	12.7
Res2	800	2.2 ± 0.6	12.2 ± 3.4	535.0 ± 2.1	24.8
Res3	700	3.0 ± 0.9	17.4 ± 5.1	478.5 ± 4.7	33.8
Res4	600	4.4 ± 1.3	26.9 ± 7.9	417.3 ± 5.2	47.7
Res5	500	7.4 ± 2.2	45.6 ± 13.3	351.2 ± 3.7	81.2
Res6	450	9.9 ± 2.9	62.0 ± 18.1	316.6 ± 2.1	104.8

### 3.3 CV at Different Mesh Resolutions

**Fig. 2.**
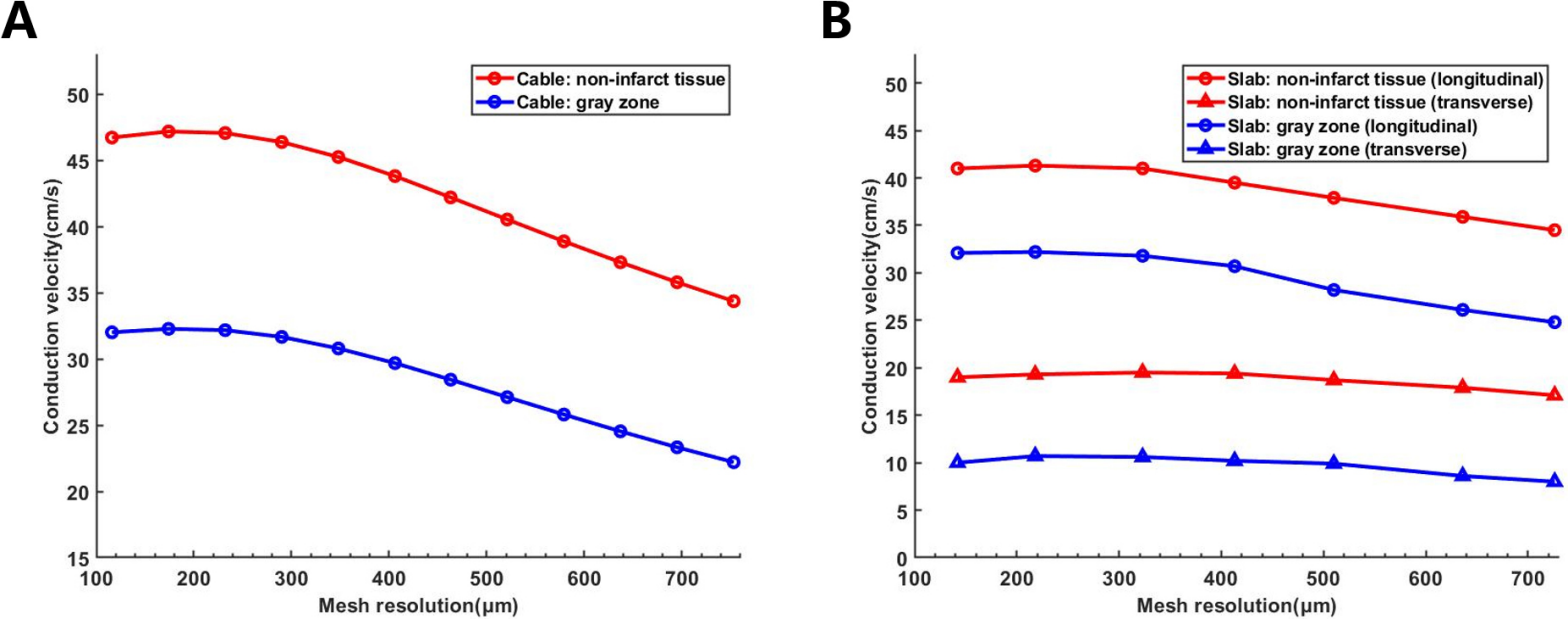
**CV variation at different mesh resolutions**. (A) CV of healthy tissue and gray zone in the cable model. (B) Longitudinal and transverse CV of healthy tissue and gray zone in the slab model. CV, conduction velocity.

For the cable simulation, the results display nonmonotonic behavior similar to that of the cuboid (Fig. 2A). The CV in healthy tissue was the fastest (47.19 cm/s) with a uniform mesh size of 150 µm (173.8 ± 30.2 µm). The maximal error of CV was 23.4% compared with the value in the finest uniform mesh size of 100 µm (115.9 ± 20.2), and the CV decreased linearly between the uniform average mesh size of 300 µm and 700 µm (red line). Similar behavior was observed in the GZ (blue line). The results of the slab simulation exhibited similar nonlinear behavior compared to the cable simulation. However, the absolute longitudinal CV decreased for healthy tissue and the GZ (Fig. 2B). The absolute and relative differences in CV were 5.1 cm/s and 12.4%, respectively, between average mesh sizes of 142 and 698 µm (red circle in Fig. 2) in the slab simulation.

Table 7 summarizes the longitudinal CV measured in the slab meshes with different resolutions. Res6 has the highest CV of 41 cm/s, whilst Res1 has the slowest CV of 35.9 cm/s for the non-infarct tissue, making the difference 5.1 cm/s. For the gray zone, the difference in CV between the two-resolution models was 5.7 cm/s, which was higher than that between the non-infarct tissues. To compensate for the lower CV caused by the coarsening of mesh resolution, the CV in models with Res1–Res5 was adjusted to match the highest-resolution CV (Table 7).

**Table 7.** **Summary of the longitudinal CV measured in slabs with different resolutions**.

Model resolution	Resolution (µm)	CV of non-infarct tissue (cm/s)	Conductivity increased	CV of GZ (cm/s)	Conductivity increased
Res1	636	35.9	18.1%	26.1	34.4%
Res2	529	37.8	16.9%	27.8	23.1%
Res3	465	38.8	11.2%	28.5	22.0%
Res4	413	39.5	7.5%	30.7	6.9%
Res5	355	40.6	5.0%	30.9	6.0%
Res6	323	41.0	-	31.8	-

CV, conduction velocity; GZ, gray zone.

Table 8 summarizes the overall simulation results and the location of the reentries induced in models with different mesh resolutions for all six patients. The VT inducibility and induced VT morphology and location were very similar in the models with resolutions from Res3 to Res6. Using Res6 as a criterion, the results in Res1 and Res2 models varied considerably.

**Table 8.** **Summary of reentries induced in models with different resolutions for all six patients**.

ID	VT in clinic	AHA location	Different model resolution					
			Res1	Res2	Res3	Res4	Res5	Res6
PAT01	N	Segment 4	-	-	-	1	-	1
PAT02	N	-	-	-	-	-	-	-
PAT03	Y	Segment 10	3	6	2	2	-	-
		Segment 7 (CR)	-	-	4	5	4	5
		Segment 5	-	-	-	-	1	-
PAT04	N	-	-	-	-	-	-	-
PAT05	N	-	-	-	-	-	-	-
PAT06	Y	Segment 7 (CR)	1	3	5	6	7	6
Accuracy			1/4 (25%)	3/9 (33.3%)	9/11 (81.8%)	11/14 (78.6%)	11/13 (84.5%)	11/12 (91.7%)

Accuracy was defined as the number of pacing site induced VT related to the clinic divide number of pacing site-induced VT. VT, ventricular tachycardia; CR, location of clinically relevant reentry induction; N, No VT detected in clinic; Y, VT detected in clinic; AHA, American Heart Association.

### 3.4 Patient-Specific Analysis: Effect of Mesh Resolution on VT

The simulation results in models with different resolutions were much more consistent for patients without clinically observed VT. No VT was induced at any sites in the models PAT02, PAT04, and PAT05 for all mesh resolutions before and after CV adjustment. For PAT01, one pacing site in the models with Res4 and Res6 induced reentry. However, the VT-inducing ratio (5.3%) was very low, suggesting that the patient had a low probability of inducing VT. For patients with clinically observed VT, the simulation results in models with different mesh resolutions were more diverse than those without clinically observed VT (Table 8).

**Fig. 3.**
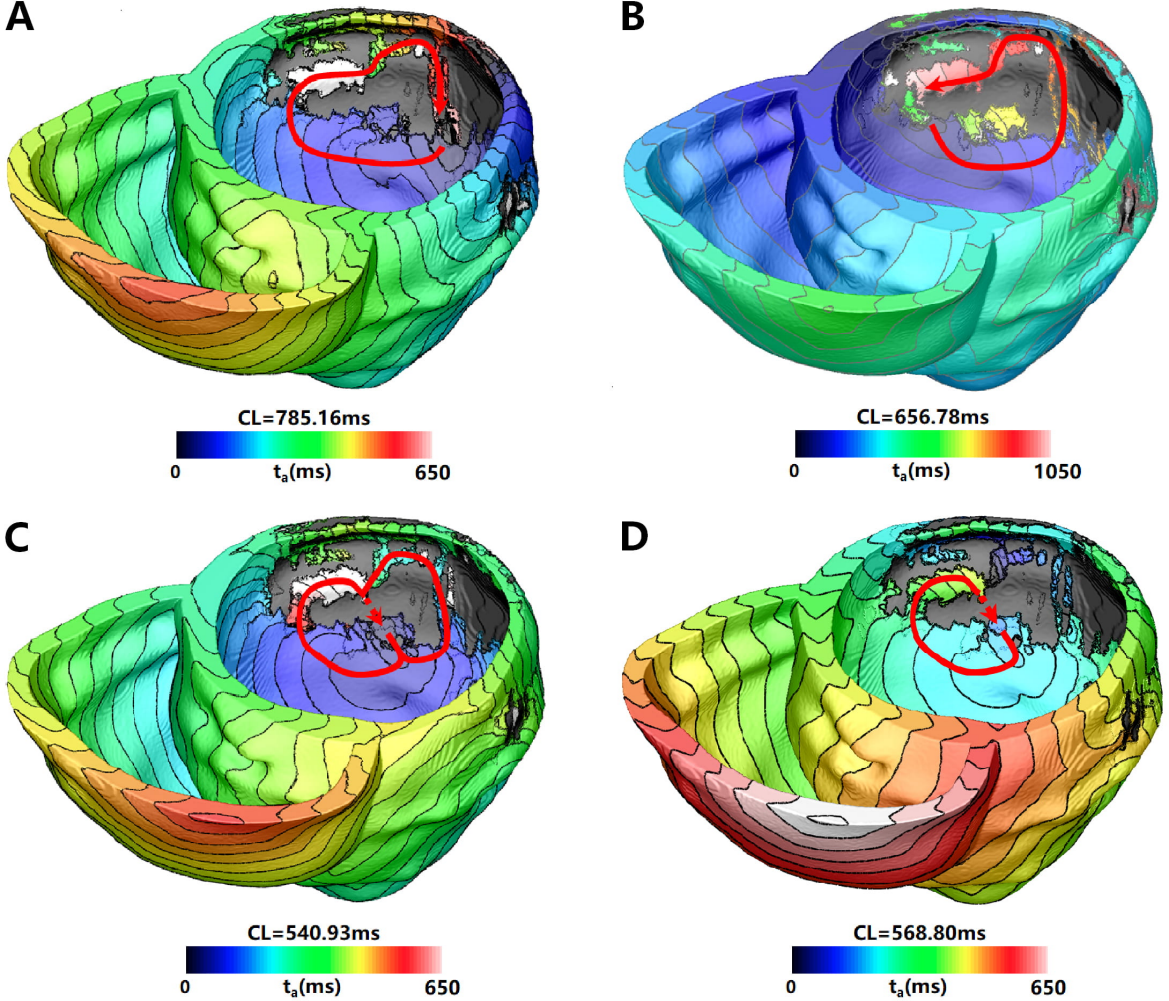
**VT morphology located at Segment 7 (clinically irrelevant) induced in simulation for PAT03 with different mesh resolutions**. VT induced in (A) Res1, (B) Res2, (C) Res3, and (D) Res4 models. The red arrowhead indicates the direction of VT propagation. The color scales in (A–D) indicate activation times, and the black areas represent the core scar (without any electrical propagation). VT, ventricular tachycardia; CL, cycle length.

In PAT03, we monitored the VT events four times in the clinic and found that the VT was located at the middle lateral wall of the LV (Segment 7 in AHA 17 segments). In the Res1 and Res2 models, three pacing sites induced a type of VT located at the posterior wall (Fig. 3A), which was unrelated to the location measured in the clinic. Though the VT induction ratio is comparable to other higher resolution models, no pacing site-induced VT related to clinical location (Fig. 3B). This VT morphology was also present in the Res3 and Res4 models (Fig. 3C,D, respectively), although at significantly smaller proportions (33.3% and 28.6%, respectively).

**Fig. 4.**
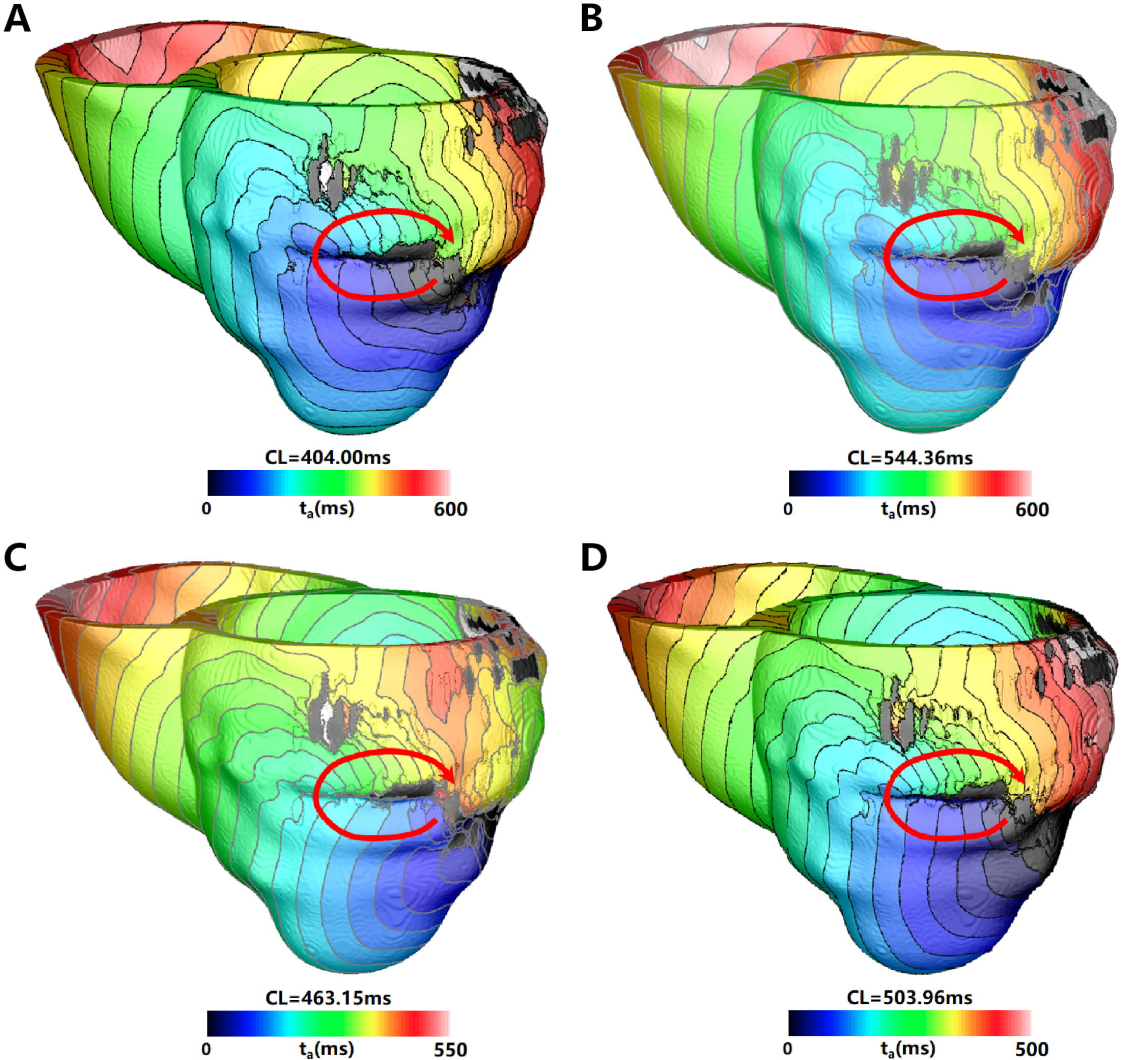
**VT located at Segment 7 (clinically relevant) induced in simulation for PAT03 with different mesh resolutions**. VT was induced in the (A) Res3, (B) Res4, (C) Res5, and (D) Res6 models. The red arrow indicates the direction of VT propagation, the color scales in (A–D) indicate activation times, and the black areas represent the core scar (without any electrical propagation). VT, ventricular tachycardia. CL, cycle length.

The results of the Res3–Res5 models are more consistent with the Res6 model (Table 8). They all induced clinically relevant VT (Fig. 4), with Res4 and Res6 inducing a maximum of five sites. Res6 did not induce any VT unrelated to the location measured in the clinic. Although Res3–Res5 had VTs unrelated to the clinical measurement, the percentage of incorrectly predicted VTs was low (20%–33.3%). When the CV was adjusted for models, the incorrectly predicted VTs were reduced in almost all models (Table 9). After the CV adjustment, Res3 was the only model with decreased accuracy. Though the incorrectly predicted VTs in segment 10 were reduced, two new incorrectly predicted VTs emerged. For the Res4 model, the number of clinical-related VTs increased from five to eight (Table 9). Although a new VT was induced at a certain pacing site that did not exist in the model with original conductivity, it cannot be considered clinically relevant owing to its low occurrence. For the Res5 model, the number of clinical-related VTs increased, while the VT unrelated to the clinical measurement disappeared. In general, the accuracy of VT prediction in the Res1, Res2, and Res3 models was not high, even after CV adjusting. Fig. 4 shows the VT morphology located at segment 7 for PAT03 with different mesh resolutions.

**Table 9.** **Characteristics of VT induced in different resolution models of patients 3 and 6 with original and modified conductivities**.

Patients	AHA location	Different model resolution										
		Res1	Res1new	Res2	Res2new	Res3	Res3new	Res4	Res4new	Res5	Res5new	Res6
PAT03	Segment 10	3	1	6	4	2	1	2	2	-	-	-
	Segment 7 (CR)	-	2	-	1	4	3	5	8	4	7	5
	Segment 5	-	1	-	-	-	2	-	1	1	-	-
	Accuracy	-	50%	-	20%	66.7%	50%	71.4%	72.7%	80%	100%	100%
PAT06	Segment 7 (CR)	1	4	3	1	5	4	6	5	7	4	6
	Accuracy	100%	100%	100%	100%	100%	100%	100%	100%	100%	100%	100%

Accuracy was defined as the number of pacing site-induced VT related to the clinic divide number of pacing site-induced VT. VT, ventricular tachycardia; CR, location of clinically relevant reentry induction; AHA, American Heart Association; CV, conduction velocity. Res(1–5)new: Simulation results with modified conductivity to match the CV of the highest-resolution model (Res6).

**Fig. 5.**
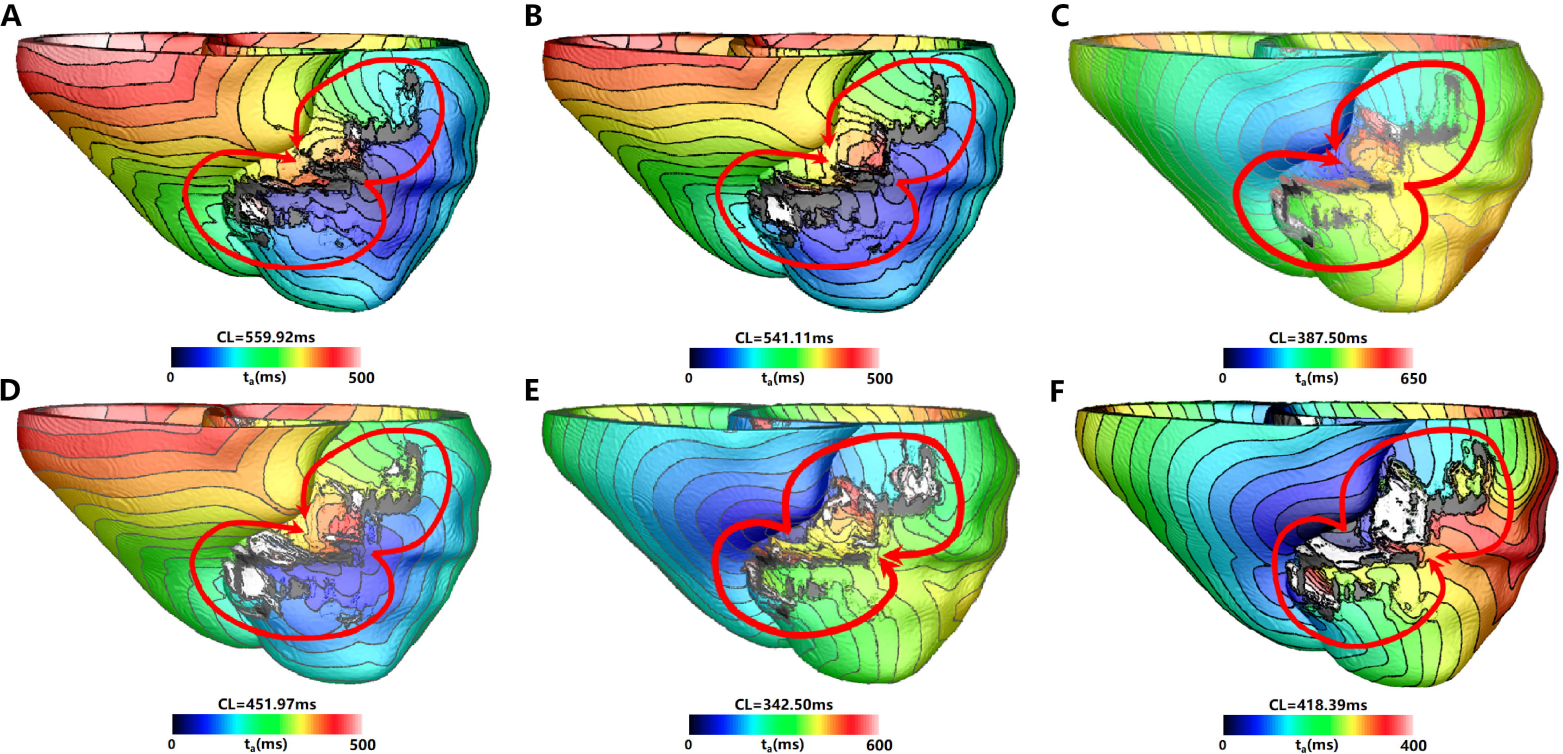
**VT morphology located at segment 7 (Clinically relevant) induced in simulation for PAT06 with different mesh resolution**. VT induced in (A) Res1 model, (B) Res2 model, (C) Res3 model, (D) Res4 model, (E) Res5 model, and (F) Res6 model. The red arrow indicates the direction of VT propagation, the color scales in (A–F) indicate activation times and the black areas represent core scar—there is no electrical propagation there. VT, ventricular tachycardia; CL, cycle length.

For PAT06, although all models with different resolutions induced clinically relevant VT (Fig. 5), the percentage of pacing sites that included VT was low for the Res1 and Res2 models (5.3% and 15.8%, respectively). However, for the Res3–Res5 models, the number of pacing sites that induced VT was very close to the highest resolution (± pacing sites). After adjusting the CV, all models simulated the same VT morphology at the same clinically relevant location. The Res2–Res5 models predicted a decrease in the number of VTs, while Res1 predicted an increase in the same (Table 9).

## 4. Discussion

In this study, we performed VT simulation studies in personalized ventricular models reconstructed from LGE–MRI images of six patients. We focused on determining the appropriate range of mesh resolution for modeling in personalized cardiac models to maintain stable arrhythmia location and morphology, as they serve as the target for VT ablation.

For our simulations, we used the full mass matrix in openCARP, which resulted in relatively stable CV measurements at different mesh resolutions. Regarding the simulation accuracy, Niederer *et al*. [19] reported that a mesh size of 0.1 mm could achieve a simulation error below 10%. If the mass matrix were used in the simulation, the simulation error with the coarse mesh size of 0.5 mm would be much smaller than if mass lumping was used [19]. Our simulation results in the cuboid showed that the relative difference in activation time at P8 with uniform tetrahedral mesh ranged from 3.5% to 70.0%. Still, this variation was reduced to a range of 0.7%–4.9% with adaptive tetrahedral mesh.

The activation time and CV results in both cuboid and cube simulations demonstrated a nonmonotone behavior when the mesh resolution was much finer (Figs. 1,2). This behavior was also reported by other groups (Figs. 8,9 in [23], Fig. 4 in [46]). Pathmanathan *et al*. [46] speculated that this was “since the CV is too fast on medium-fine meshes (interpolated ionic current too large) and too slow coarse meshes (magnitude of ionic current not resolved), there is a crossover point where these errors balance each other, and the CV is correct”.

Although the analysis of activation time and CV in the cuboid and cube simulations showed convergence and below 10% error for models with adaptive tetrahedral mesh resolution at an average resolution below 600 µm, simulation results in our patient-specific models revealed different conclusions. Simulation results in the six different mesh resolution models indicated that the safe threshold for mesh size in patient-specific models should be below 600 µm.

The simulation was numerically convergent in the Res2 models, but none of the pacing sites induced clinical VT. Even with conductivity adjustment, the induced clinical VT ratio was very low (20%). For the Res3 models, 66.7% of the pacing sites induced clinical VT. When conductivity was adjusted to match the model with the highest mesh resolution, the pacing sites with positive predicted VT decreased, while those with false predicted VT increased. Thus, we do not recommend the Res3 models, even though they can achieve numerical convergence.

Although the Res4 model had false predicted VT, the pacing sites that induced these false VT were relatively small (<30%). When conductivity was adjusted to match the model with the highest resolution, the pacing sites with positive predicted VT increased substantially, although one extra false VT emerged. The falsely predicted VTs had a much lower inducible probability, making them clinically irrelevant. Thus, while the Res4 model is not ideal, it can predict VT if the VTs induced in personalized cardiac models are prioritized and only high-priority VTs are considered positive predicted VTs.

In the Res5 models, 80% of the pacing sites induced clinically relevant VT. When conductivity was adjusted to match the model with the highest mesh resolution, the pacing sites with correct prediction increased to 100%. For the Res6 models with the finest mesh resolution, VT inducibility, and location were accurately predicted for all four patients. The average mesh resolution used in personalized heart models typically falls within the range of 350–400 µm (350 µm in [12], 400 µm in [27], 390 µm in [26], 350 and 400 µm in [28]), consistent with current findings suggesting that mesh resolutions of Res4 or finer can predict most clinical VTs.

Undoubtedly, the models with the highest resolution offer the greatest accuracy and lowest false VT prediction, resulting in the best match with clinical results among the selected resolutions. However, even a small difference in spatial discretization between models with different resolutions (an average difference of 55 µm) can substantially increase the simulation time in each model (Table 6), exponentially increasing the simulation time. Given that patient-specific VT simulation typically takes less than 48 hours from MRI image processing to obtaining final simulation results, the Res6 models cannot meet clinical demands. To comply with clinical time constraints, we utilized the Res4 models in our previous article [13], which was proven to be accurate for those models in this study.

The accuracy of VT prediction based on personalized cardiac simulation is influenced by many factors. Not only the size of the spatial resolution involved in this paper but also other factors, including electrophysiological cell models, numerical solvers, and grid types [19], which can cause large variability in the final simulation results or the choice of conductivity in the longitudinal and transverse direction, etc. This is something that needs to be critically analyzed and discussed. The fiber orientation, single-cell models, and the conductive were already validated by other groups or our previous studies [12, 13, 25, 36]. Deng *et al*. [47] analyzed the sensitivity of ablation targets to electrophysiological parameter variability. They reported that VT ablation target uncertainty in patient-specific ventricular models with an average representation of VT-remodeled electrophysiology is relatively low. Personalized ventricular modeling with an average representation of infarct-remodeled electrophysiology may uncover most targets for VT ablation. Furthermore, the VT location predicted by us matched the clinic measurements for nearly all patients whenever VT was detected in the clinic, indicating that our method and parameters can predict the reentry observed in the clinic.

The primary findings of this study about clinical applications of computational modeling are as follows: (1) Both full mass matrix and adaptive tetrahedral mesh enable cardiac tissue electrophysiology simulations to maintain a broader range of mesh sizes while achieving below 10% error rate. Most theoretical accuracy studies of cardiac tissue electrophysiology simulations rely on uniform tetrahedral mesh, requiring mesh resolutions of at least 0.1 mm [19, 23] or 0.25 mm for convergence [18]. (2) Average mesh resolutions below 350 µm can attain an accuracy of over 85% for clinically relevant VT. When conductivity is adjusted to match the CV in the model with the finest mesh size, the overall ratio of positively predicted VTs increases.

### Limitations and Future Directions

A limitation of this study is the small sample size, consisting of only six patients. This is due to the challenges in obtaining good-quality MRI images and patient follow-up data. Despite the small patient dataset, we believe the conclusion remains unaffected. Another limitation is that the smallest mesh size is approximately 300 µm, a constraint imposed by the 64GB memory capacity of the computer utilized for model generation. As the simulation results in cable and patient-specific models with Res5 and Res6 exhibited no significant differences, we believe that models with resolutions higher than 300 µm will not be adversely affected.

## 5. Conclusions

Here, we examined the influence of finite element mesh size on the accuracy of VT prediction using personalized virtual heart simulation. We found that a personalized heart model can optimally balance the simulation time and VT prediction accuracy when discretized with an average edge length of approximately 350 µm for the tetrahedral mesh. When the CV is adjusted, incorrect VTs caused by excessive mesh resolution can be effectively reduced, and VTs that align with clinical findings can be improved.

## Data Availability

The original contributions presented in the study are included in the article material, further inquiries can be directed to the corresponding author/s.
